# Psychological Disorders among Iranian Infertile Couples Undergoing Assisted Reproductive Technology (ART)

**Published:** 2017-03

**Authors:** Mansoureh KARIMZADEH, Nasser SALSABILI, Firouzeh AKBARI ASBAGH, Robab TEYMOURI, Golamreza POURMAND, Tahereh SOLEIMANIEH NAEINI

**Affiliations:** 1. Pediatric Neurorehabilitation Research Center, University of Social Welfare and Rehabilitation Sciences, Tehran, Iran; 2. Dept. of IVF, Mirza Koochak Khan Hospital, School of Rehabilitation, Tehran University of Medical Sciences, Tehran, Iran; 3. Dept. of Obstetrics and Gynecology, School of Medicine, Tehran University of Medical Sciences, Tehran, Iran; 4. Urology Research Center, Sina Hospital, School of Medicine, Tehran University of Medical Sciences, Tehran, Iran

**Keywords:** Infertility, Psychological disorders, Assisted reproductive technology (ART)

## Abstract

**Background:**

Worldwide, infertility affects 10%–15% of couples and most of them seek medical help including Assisted Reproductive Technology (ART) treatments. Undergoing ART treatments create many physical and emotional burdens. This study examined the psychological consequences of infertility in Iranian infertile males and females as well as their spouses, unlike previous studies that examined mainly females with infertility.

**Methods:**

Subjects in this descriptive analytical design were recruited from the IVF Department of Mirza Koochak Khan Hospital and the Rouyesh Infertility Treatment Center of Tehran, Iran between Aug 2014 and Sep 2015. Overall, 256 couples (64% response rate), consisting of 78 infertile male and their spouses and 50 infertile female and their spouses, were included in this research. The psychological disorders were measured by the Persian version of Symptoms Checklist-90-Revised and Cattle Inventory.

**Results:**

Psychological disorders of infertile couples are significantly associated with increasing age, higher education, longer duration of infertility and unemployment (*P*<0.05). Prevalence of anxiety, depression, hypochondriasis and paranoia in infertile females and spouses of infertile males were significantly higher than husbands of infertile females (*P*<0.05). Obsession was more sever in infertile females was significantly greater than infertile males (*P*=0.01). Depression was significantly lower in infertile males than their spouses (*P*=0.016).

**Conclusion:**

Iranian infertile females and spouses of infertile males experienced more psychological disorders than infertile males and spouses of infertile females. These results may be due to the impact of cultural beliefs and gender roles in Iranian society. Anxiety, depression, obsession, paranoia and hypochondriasis should be addressed before any ART treatments.

## Introduction

Infertility is a serious and prevalent health problem in developing countries (1). It inflicts 10%–15% of couples trying for a child (2, 3) and the trend is increasing in many societies and industrialized countries (4). It is defined as the unsuccessful attempts to attain a pregnancy after one year or more of unprotected intercourse (5). The causes of infertility may be categorized into three main groups: 1) the male factors, 2) the female factors and 3) both female and male factors or unknown causes (6). About 30%–40% of factors were related to males. The possible risk factors in females included tubal and peritoneal pathology (30%–40%), ovulatory dysfunctions (15%). The remaining 5% was attributed to uterine pathology and other unknown causes (6). Several factors affecting fertility rates involve the enhanced presence of females in employment and higher education, partnership’s inconsistency, delayed childbearing, values’ change and increasing economic difficulties (7). In Iran, 78.4% of the couples suffer from primary infertility and 21.6% suffer from secondary infertility. Percentage distribution of gender infertility contribution was 34.0% in male factor, 43.5% in female factor, 17.1% in both male and female factors and the remaining 8.1% was related to unknown causes (8).

Couples suffering fertility problem often seek medical help including assisted reproductive technology (ART) treatments (9). During the past 10 years, the number of females treated with ART had raised (10). An estimated 5 million babies and more had been born throughout the world ever since the first successful IVF baby was delivered (11). Thus, many infertile couples have been found treatment with ART helpful (12). However, undergoing ART treatments do create many physical, economic and emotional burdens (13–15). Several studies have examined the psychological consequences associated with infertility treatments (15–37). Some of them have found correlations between distress, mostly anxiety, and depression, and low ART treatment outcomes (32–38), However, a handful of studies had not reported such correlations (39–42).

In addition, psychological problems of infertile couples are correlated with age, employment, education, duration of infertility and gender. For example, depression among infertile females was directly related to age and longer duration of infertility (19, 22, 24, 43). Infertile housewives were exposed to a higher risk of developing psychiatric disorders than working infertile females (15). Infertility could be a very stressful and distressing experience, particularly in females (38, 44–49). Females will be more affected by the social burden of the treatment and less affected by any changes in the interpersonal functioning of the couples; while husbands were more concerned about the couples’ relationship and interpersonal strategies rather than the cost of the treatment (50).

Although there are many studies on psychological distress among females with infertility (10, 13, 15, 16, 19, 21, 29, 31–33, 37, 51, 52), studies examining both partners’ psychological distress status in infertile couples have not been reported.

This study examined the psychological impact of infertility in both Iranian infertile males and females as well as in their spouses.

## Materials and Methods

### Participants

A total of 256 couples, consisting of 78 infertile male (IM) and their spouses and 50 infertile female (IF) and their spouses, were included in this analytical and descriptive study from 400 couples undergoing ART treatment at the IVF department of Mirza Koochak Khan Hospital and the Rouyesh Infertility Treatment Center, Tehran, Iran. Couples referred to two centers between Aug 2014 and Sep 2015 got both verbal and written detailed information about the study.

The couples were recruited by convenience sampling during the study period. The inclusion criteria were: 1) willingness to participate in the study and 2) ability to complete independently the study questionnaires. Patients were excluded if they used treatment other than IVF and ICSI such as IUI and had not completed the questionnaire.

### Procedure

The questionnaires package was given to the couples who agreed to participate in the study. Informed written consents were obtained from all couples. Demographic information like age, gender, education and duration of infertility were included.

The study was approved by the Research Ethics Committee of the University of Social Welfare and Rehabilitation sciences (via approval No. USWR.REC.239 Dated July 2014), Tehran, Iran.

### Measurements The Symptom Checklist-90-Revised (SCL-90-R)

Leonard R. Derogatis (1970s) developed the SCL-90-R. It is a self-report symptom inventory to measure psychological symptoms and distress (53). It contains 90 items using a five-point scale, with responses ranging from zero (not at all) to four (extremely). The SCL-90-R assesses psychological distress in terms of nine primary symptom dimensions. Somatization, Interpersonal Sensitivity, Obsessive-Compulsive, Depression, Anxiety, Hostility, Paranoid Ideation, Phobic Anxiety, and Psychoticism are the nine dimensions. The internal consistency reliability for the 9 subscales was 0.36–0.73 in SCL-90-R (53).

In this study, the Persian translated version of Symptoms Checklist-90-Revised (SCL-90-R) was used. The Cronbach’s Alpha coefficient for the Persian version was 0.72 and its’ inter-rater reliability was 0.80. The participants are to answer the questions. Then total scores derived from the Persian version of SCL-90-R. An average of ≥1 and ≥3 represent psychological disorder, and psychotic and depression, respectively.

### The Cattle Inventory (CI)

The CI is a self-report measure of anxiety. It contains 40 items using a three-point scale, with responses ranging from zero to two. Scores were ranged from zero to 80, with scores of 28 or more indicating anxiety. Classification of anxiety scores involves 0–27 (without anxiety), 28–40 (moderate anxiety), 41–49 (neurotic anxiety) and 50–80 (severe anxiety). The CI utilized in this study is Iranian validated version (54).

### Statistical Analysis

To describe the general characteristics of the participants, descriptive statistics (e.g., mean, standard deviation and percentages) were used. Multiple regression analysis and multivariate analysis of variance (MANOVA) were conducted on the research data from Iranian infertile couples with Statistical Package for Social Sciences (SPSS) version 19.0 (Chicago, IL, USA). The alpha level was 0.05. Normality of the data distribution was determined.

## Results

Of the 400 couples, 144 cases had not completed the questionnaires. Their data were excluded from the final analyses. This led to 256 couples in the final sample. The mean age of IM, spouses of IM, IF and spouses of IF in this study were 31.68 yr (±3.43), 28.42 yr (±4.87), 28.3 yr (±5.96) and 33.37 yr (±5.98), respectively. The majority of the participants had high level of university education (63.92%) (Table 1).

**Table 1:** Demographic characteristics of study participants (n=256)

**Characteristic**	**Infertile female (IF)**			**Spouses of IF**			**Infertile male (IM)**			**Spouses of IM**		
	**N**	**Mean**	**SD**	**n**	**Mean**	**SD**	**n**	**Mean**	**SD**	**n**	**Mean**	**SD**
Age, Mean (SD)	50	28.3	5.96	50	33.37	5.98	78	31.68	3.43	78	28.42	4.87
*Education*												
Lower Diploma	26	82%		19	36.8%		22	28.2%		26	33.3%	
Higher Diploma	24	48%		31	63.2%		56	71.8%		52	66.7%	
Duration of infertility per year (SD)		5.9 (4.19)		5.9 (4.19)			5.5 (2.76)			5.5 (2.76)		
*Psychological Variables*												
Anxiety (SCL-90-R)	50	1.6	1.15	50	1.17	0.68	78	1.3	0.75	78	1.41	0.82
Depression	50	1.44	0.94	50	0.95	0.71	78	1.18	0.73	78	1.55	0.69
Hypochondriasis	50	1.36	0.83	50	1.24	0.63	78	1.33	0.73	78	1.37	0.67
Obsession	50	1.57	0.90	50	1.03	0.69	78	1.16	0.73	78	1.42	0.73
Compulsion	50	1.43	0.83	50	0.92	0.8	78	1.26	0.78	78	1.33	0.62
Aggression	50	1.32	0.89	50	1.01	0.85	78	1.19	0.86	78	1.25	0.92
Paranoia	50	1.71	0.89	50	1.2	0.72	78	1.57	0.77	78	1.7	0.79
Phobia	50	1.3	1.36	50	0.84	1.1	78	0.81	0.84	78	0.86	0.82
Psychotics	50	1.17	0.79	50	0.83	0.7	78	1.11	0.83	78	1.2	0.69
Anxiety (CI)	50	33	7.5	50	29	4.9	78	32	6.5	78	34	6.1

The mean score of psychological symptoms (SCL-90-R) among four groups was greater than one except for depression, compulsion, phobia and psychotics in spouses of IF and phobia in IM and their spouses (an average of ≥1 represents psychological disorder). The findings of the Cattle Inventory indicated that anxiety was moderate in all four groups.

The psychological disorders were examined in four groups by using MANOVA test (Table 2). Anxiety, depression, hypochondriasis and paranoia in IF and spouses of IM were significantly higher than spouses of IF (*P*<0.05). Depression in spouses of IM was higher than IM (*P*=0.016). Obsession in IF was greater than IM (*P*=0.01). Anxiety and paranoia in IM were greater than spouses of IF (*P*=0.035, *P*=0.009) (Table 2).

**Table 2:** Effects of psychological variables (SCL-90-R) between infertile groups and their spouses

**Variables**	**Group1**	**Group2**	**Mean Difference**	**SD**	***P*-value**
Anxiety	Spouses of IF	IM	−3.12	1.149	0.035
		Spouses of IM	−5.21	1.149	0.0001
		IF	−4.31	1.267	0.004
	IM	Spouses of IF	3.12	1.149	0.035
Depression	Spouses of IF	Spouses of IM	−0.59	0.139	0.001
		IF	−0.59	0.139	0.001
	IM	Spouses of IM	−0.36	0.123	0.016
Obsession	Spouses of IF	Spouses of IM	−0.38	0.139	0.029
		IF	−0.54	0.159	0.003
	IM	IF	−0.42	0.138	0.01
Hypochondriasis	Spouses of IF	Spouses of IM	−0.412	0.136	0.014
		IF	−0.51	0.156	0.014
Paranoia	Spouses of IF	IM	−0.458	0.144	0.009
		Spouses of IM	−0.50	0.144	0.003
		IF	−0.59	0.144	0.001

MANOVA test was used for comparison among four groups

### Infertile Male (IM)

There was significant correlation between depression and anxiety in infertile males (*P*=0.0001, R=0.531) (Table 3). Stepwise regression analysis indicated that higher education significantly predicted 18% of anxiety (*P*=0.0001), 8% of depression (*P*=0.034) and 22% of paranoia (*P*=0.0001) (Table 4). In addition, increasing age significantly identify 8.2% of obsession (*P*=0.032) and 12% of paranoia (*P*=0.00007) (Table 4).

**Table 3:** Results of stepwise regression analysis of anxiety by psychological variables (SCL-90-R) among four groups

**Groups**	**Criterion Variable**	**Regression Variables**	**R (β)**	**R^2^**	***P*-value**	**F**
Infertile male (IM)	Anxiety	Depression	0.531	0.281	0.0001	29.8
Spouses of IM	Anxiety	Paranoia	0.36	0.13	0.0001	11.33
Infertile female (IF)	Anxiety	Psychotics	0.51	0.256	0.0001	16.5
Spouses of IF	Anxiety	Hypochondriasis	0.311	0.097	0.029	5.48
		Phobia	0.501	0.251	0.0001	7.71
		Depression	0.56	0.313	0.0001	6.83

**Table 4:** Results of stepwise regression analysis of psychological variables (SCL-90-R) in infertile groups and their spouses

**Groups**	**Criterion Variable**	**Regression Variables**	**R (β)**	**R^2^**	***P*-value**	**F**
Infertile male (IM)	Anxiety	Education	0.433	0.187	0.0001	12.44
	Depression	Education	0.284	0.08	0.034	4.75
	Paranoia	Education	0.469	0.22	0.0001	7.5
	Obsession	Age	0.286	0.082	0.032	4.8
	Paranoia	Age	0.357	0.127	0.00007	7.88
Spouses of IM	Obsession	Age	0.301	0.091	0.032	4.88
	Hypochondriasis	Age	0.28	0.079	0.046	4.18
	Psychotics	Age	0.297	0.088	0.034	4.75
Infertile female (IF)	Anxiety	Duration of Infertility	0.432	0.187	0.022	5.97
	Depression	Duration of Infertility	0.506	0.256	0.0006	8.96
	Psychotics	Age	0.576	0.332	0.00006	6.22
	Anxiety	Age	0.607	0.37	0.00003	7.31
	Aggression	Age	0.451	0.204	0.00006	6.64
	Psychotics	Employment	0.389	0.151	0.04	4.63
	Anxiety	Employment	0.539	0.291	0.014	5.13
	Paranoia	Employment	0.523	0.273	0.0004	9.78
	Hypochondriasis	Employment	0.512	0.262	0.0005	9.24

### Spouses of IM

There was significant correlation between paranoia and anxiety in spouses of IM (*P*=0.0001, R=0.36) (Table 3). Stepwise regression analysis showed that increasing age significantly predicted 9% of obsession (*P*=0.032), 7.9% of hypochondriasis (*P*=0.046) and 8.8% of psychotics (*P*=0.034) (Table 4).

### Infertile Female (IF)

There was significant correlation between psychotics and anxiety in IF (*P*=0.0001, R=0.51) (Table 3). Stepwise regression analysis indicated that 18% of anxiety (*P*=0.022) and 25% of depression (*P*=0.0006) have been predicted by duration of infertility. Increasing age significantly identify 33% of psychotics (*P*=0.00006), 37% of anxiety (*P*=0.00003) and 20% of aggression (*P*=0.00006). In addition, employment significantly predicted 15.1% of psychotics (*P*=0.04), 29.1% of anxiety (*P*=0.014), 27.3% of paranoia (*P*=0.0004) and 26.2% of hypochondriasis (*P*=0.0005) (Table 4).

### Spouses of IF

There was significant correlation between anxiety and hypochondriasis in spouses of IF (*P*=0.029, R=0.31), phobia (*P*=0.0001, R=0.501) and depression (*P*=0.0001, R=0.56) (Table 3).

## Discussion

Reproduction is one of the vital and natural human goals for survival of every society and when childbearing seems impossible, probable psychological crisis begins (55). This study showed that Iranian infertile females and spouses of infertile males experienced more psychological disorders than infertile males and spouses of infertile females. Anxiety, depression, hypochondriasis and paranoia in infertile females and spouses of infertile males were significantly higher than husbands of infertile females. Obsession in infertile females was higher than infertile males. Depression was significantly lower in infertile males than their spouses were. Thus, in Iranian society, husbands of infertile females may have extended coping abilities to better control the concerns of their situation. While females cope with infertility less well, even if they were not the source and cause of infertility. Cultural beliefs, gender roles, and traditional society may be important risk factors for infertile couples psychologically. Our results concurred with previous studies examining females bearing the major burden of infertility psychologically, even when they know there is a male cause (56–58). These results thus expand previous findings pertaining to infertility and psychological consequences of infertility (44–49).

In addition, higher education was associated with increased anxiety and depression in infertile males. Anxiety and depression in infertile females were worsened by increasing age and longer duration of infertility. Our study results are in accordance with previous studies (19, 22, 24, 43, 59, 60). Increasing age resulted in increased obsession and hypochondriasis in infertile males and increased psychotics and aggression in infertile females. Increased anxiety and psychotics are associated with unemployment in infertile females. Our result is consistent with other study in Iran (15).

Previous studies have examined enhanced risk of depression, anxiety, mood disorders and psychiatric disorders mainly in females with infertility (10, 15, 20, 21, 35–37). Until recently, most studies are limited to clinic samples seeking and receiving treatment. The enhanced risk indicated major diversity probably because of considerable differences in methodology and study design. Our study design varies from previous studies since most studies have not compared infertility problems of Iranian infertile females and males according to their spouses’ status.

This study had some limitations. First, since the researchers had relied on convenience samples drawn from only two clinic samples and not a multi-central trial, generalizing to entire Tehran city may be limited. Second, because it is drawn from only Iranian couples, generalizing to other ethnic minority and even non-city dwellers may be limited.

## Conclusion

Regardless of infertility factors, females were more vulnerable to psychological disorders. Childbearing inability was always attributed to women. They were blamed and held responsible for infertility even if they are not the cause. These results may be due to differences in cultural beliefs in Iranian society, as well as the gender roles of Iranian females compared to males. It had been suggested that psychological counseling and cognitive behavioral therapy (CBT) prior to ART treatment might help to reduce psychological distress in couples undergoing infertility treatment and increase the possibility of pregnancy. Future studies should investigate how the recommended psychological therapies are effective in reducing psychological distress in couples undergoing ART treatment as well as how these interventions affect pregnancy rate. The other area that needs further research is the psychological profile of non-treatment seekers and non-clinic samples in bigger scale.

## Ethical considerations

Ethical issues (Including plagiarism, informed consent, misconduct, data fabrication and/or falsification, double publication and/or submission, redundancy, etc.) have been completely observed by the authors.
